# Serum triglyceride concentrations and cancer risk in a large cohort study in Austria

**DOI:** 10.1038/sj.bjc.6605264

**Published:** 2009-08-18

**Authors:** H Ulmer, W Borena, K Rapp, J Klenk, A Strasak, G Diem, H Concin, G Nagel, VHM & PP Study Group

**Affiliations:** 1 Department of Medical Statistics, Informatics and Health Economics, Innsbruck Medical University, Innsbruck, Austria; 2 Institute of Epidemiology, Ulm University, Ulm, Germany; 3 Agency for Preventive and Social Medicine, Bregenz, Austria

**Keywords:** STGs, cancer incidence, epidemiology

## Abstract

**Background:**

Blood lipid levels as part of the metabolic syndrome are thought to be linked to cancer risk. Few epidemiological studies have addressed the association between serum triglyceride (STG) concentrations and cancer risk.

**Methods:**

Serum triglyceride concentrations were collected in a health investigation (1988–2003). The analyses included 156 153 subjects (71 693 men and 84 460 women), with 5079 incident cancers in men and 4738 cancers in women, and an average of 10.6 years of follow-up. All malignancies were ascertained from the population cancer registry. Multivariate Cox proportional hazard models stratified by age and sex were used to determine adjusted cancer risk estimates and 95% confidence interval (95% CI).

**Results:**

In men and women combined, higher STG concentrations were associated with increased risk of lung (4th *vs* 1st quartile: HR, 1.94; 95% CI, 1.47–2.54), rectal (HR, 1.56; 95% CI, 1.00–2.44), and thyroid cancer (HR, 1.96; 95% CI, 1.00–3.84). Serum triglyceride concentrations were inversely associated with non-Hodgkin's lymphoma. In men, STG concentrations were inversely associated with prostate cancer and positively with renal cancer. In women, STG concentrations were positively associated with gynaecological cancers. Stratification by BMI revealed a higher risk of gynaecological cancers in overweight than in normal weight women. No other associations were found.

**Conclusions:**

Our findings support the hypothesis that STG concentrations are involved in the pathogenesis of lung, rectal, thyroid, prostate, and gynaecological cancers.

Obesity has been identified as a major risk factor for such cancer sites as colon, renal, breast, and endometrium (Bianchini *et al*, 2002; Calle and Kaaks, 2004; Rapp *et al*, 2005), whereas hypertriglyceridemia is relevant to obesity and insulin resistance (Despres and Lemieux, 2006). Dietary fat intake is a well-established risk factor in cardiovascular diseases (CVDs), in which much investigation has involved serum triglyceride (STG) concentrations (Sarwar *et al*, 2007). The combination of hypertriglyceridemia and elevated waist circumference has been identified as a phenotype for higher risk of CVD (Kahn and Valdez, 2003). Usually, fasting triglyceride concentrations are measured, as they are associated with increased mortality and CVD risk (Brunzell, 2007). However, there is uncertainty with regard to the impact of STG concentrations on risk of CVD (Gotto, 1998 and also with regard to whether fasting level influences the relationship (Langsted *et al*, 2008). Beyond lipid metabolism there is evidence that hypertriglyceridemia is associated with frequent infections and inflammation (Khovidhunkit *et al*, 2004; Esteve *et al*, 2005).

A few cohort studies have investigated high STG concentrations as a part of the metabolic syndrome (Tulinius *et al*, 1997) in relation to risk of colon (Saydah *et al*, 2003; Ahmed *et al*, 2006, Tande *et al*, 2006), breast (Vatten and Foss, 1990; Furberg *et al*, 2004), and cervix cancers (Cust *et al*, 2007). A cohort study among Icelanders (Tulinius *et al*, 1997) revealed associations between high STG levels and colorectal cancers in both sexes, and also with thyroid cancer in men, as well as with cervix, endometrial, and bladder cancer in women (Tulinius *et al*, 1997).

We therefore investigated the associations between fasting STG concentrations and cancer risk in a large prospective cohort study.

## Methods

### Study population

Details of the Vorarlberg Health Monitoring and Promotion Program (VHM&PP) in Vorarlberg, the most western region in Austria, are provided elsewhere (Rapp *et al*, 2005). In brief, the Agency of Social and Preventive Medicine annually offered to all adults living in Vorarlberg a screening examination that includes a physical examination, a blood test, and a consultation with a doctor. By 2005, ∼56% of all Vorarlberg residents underwent at least one examination in this voluntary screening programme. Between 1988 and 2003, over 156 000 adult Vorarlberg residents were enrolled in the cohort after signing an informed consent form to store and process personal data and biological samples.

For the current analysis, we used a data set with complete data on STG and covariates at baseline. Participants with follow-up <1 year (*n*=6188), or with prevalent cancer (other than non-melanoma skin cancer), were excluded before enrolment or within 1 year after enrolment (*n*=2149).

Two central laboratories, with regular internal and external quality tests, determined STG concentrations on fasting blood samples. Within 60–240 min of venous blood sample collection from a cubital vein, serum was obtained by centrifugation for 15 min at 4000 r.p.m. Subsequently, STG concentrations were measured at 37°C and were expressed as mg per 100 ml. To check calibration, three daily control samples were included. If average values of control samples of each run were not within 3% of the true value, the run was repeated. Day-by-day variation had to be within 5%. Study participants are classified according to the quartiles of STG concentrations with the following cutoff values: ⩽83, 84–119, 120–179, and ⩾180 mg 100 ml for men and ⩽69, 70–94, 95–133, and ⩾134 mg 100 ml for women. Participants in the 1st quartile were used as reference category.

Measurements of height, weight, blood pressure, total cholesterol, blood glucose, and gamma-glutamyltransferase (GGT) were obtained routinely for each participant. BMI was calculated by height and weight at baseline and was categorised on the basis of clinical guidelines (<25 kg m^–2^, 25 to <30 kg m^−2^, ⩾30.0 kg m^−2^) (World Health Organisation, 1998). Smoking status was classified as current, former, or non-smokers. Participants who never smoked could not be distinguished from those who did not respond to questions with regard to smoking at baseline, but baseline smoking status was verified for >70% of study participants on the basis of information provided at subsequent examinations. As a proxy for socioeconomic position, the occupational group (blue collar, white collar, or self-employed) was determined by the participant's insurance number. Retired participants were classified according to their former occupation, and housewives on the basis of the job of their spouse.

As described previously in detail (Rapp *et al*, 2005), cancer cases were identified by record linkage with the Vorarlberg cancer registry, which has been accepted for IARC publication since 1993 (Parkin DM *et al*, 2003) and has high completeness of ascertainment (Oberaigner W, 2006). In the Vorarlberg cancer registry, nearly all cancers (96.7%) were histologically verified and the Death-certificate-only (DCO) rate meets international quality criteria (5% for both sexes in 1998–2002). Cohort data were linked to the Vorarlberg Death Index to identify deaths and to calculate person-years. The current analysis makes use of the data set updated at the end of 2003. The average follow-up time was 10.6 (s.d. 4.5) years. The 10th Revision of the *International Statistical Classification of Diseases, Injuries and Causes of Death* (ICD) was used to code the cancers (World Health Organization, 2008).

### Statistical analysis

The analytical cohort comprised 156 153 subjects (71 693 men and 84 460 women). Partial correlation coefficients were calculated to examine the relationship between STG and other clinical parameters. Cox proportional hazard models were used to compute hazard ratios (HRs) and 95% confidence intervals (CIs) for quartiles relative to the reference group (1st quartile of STG level). Models were adjusted for serum concentrations of glucose (mg per 100 ml, continuous)(Rapp *et al*, 2006), total cholesterol (mg per 100 ml, continuous) (Ulmer *et al*, 2004; Strasak *et al*, 2009), GGT (U l^–1^, continuous) (Strasak *et al*, 2008a,2008b), body mass index (BMI, kg m^−2^, continuous) (Rapp *et al*, 2005), occupational status, and smoking status (both in classes). Continuous risk estimates are presented for an increase in exposures of one unit log-transformed STG concentration. To test the overall significance of exposure, *P*-values for Wald *χ*^2^ statistics are shown. As no obvious sex differences between the estimates emerged, models were calculated for the sexes combined. All *P*-values are two-sided and all calculations were carried out with SAS statistical software package SAS release 9.1 (SAS Institute, Cary, NC, USA).

## Results

During follow-up, 5079 incident invasive cancer cases among men and 4738 incident invasive cancer cases among women were identified (Table 1). Correlations between BMI, age, and serum concentrations of STG, total cholesterol, glucose, and GGT are shown in Table 2. STG was weakly associated with serum glucose concentrations and moderately associated with BMI, total cholesterol, and GGT concentrations.

Table 3 shows the hazard ratios for cancer type by STG concentrations in the VHM&PP cohorts. Compared with the 1st quartile, high STG concentrations (4th quartile) were associated with increased risk of lung (HR, 1.94; 95% CI, 1.47–2.54), rectal (HR, 1.56; 95% CI, 1.00–2.44), and thyroid cancer (HR, 1.96; 95% CI, 1.00–3.84). High STG concentrations were inversely associated with non-Hodgkin's lymphoma. Prostate cancer was inversely associated with STG concentrations (per log-unit HR, 0.80; 95% CI, 0.72–0.90) and was positively associated with incidence of kidney cancer in men (data not shown). High STG concentrations were associated with higher overall cancer risk (4th *vs* 1st quartile: HR, 1.19; 95% CI, 1.05–1.33) and with risk of gynaecological cancers (endometrium, ovar, cervix) (4th *vs* 1st quartile: HR, 1.62; 95% CI, 1.13–2.33).

**Figure 1:**
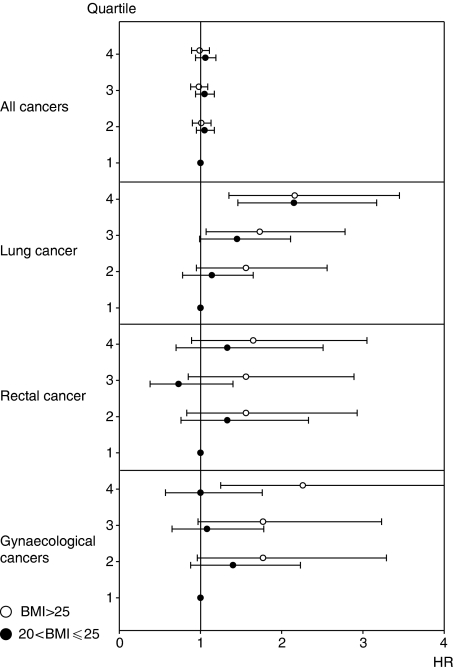
Incidence of selected cancer sites according to sex-specific quartiles of serum triglyceride concentrations in the study population (*N*=156 153) by BMI^*^. ^*^Adjusted for BMI (kg m^−2^, continuous), GGT (continuous), serum glucose (continuous), total cholesterol concentration (continuous), smoking status, occupational status, and sex (not for gynaecological cancers).

Figure 1 shows HRs for selected cancers by STG concentrations stratified by BMI. These did not reveal differential associations of STG levels with cancer overall, or with lung and colon cancer risk; however, the risk of gynaecological cancers was higher in overweight than in normal weight women. When data were stratified by smoking status (data not shown), no differential estimates emerged for overall and gynaecological cancers, but a somewhat higher risk of rectal cancer was found in current smokers (*N*=50 cases; per log-unit HR, 1.73; 95% CI, 1.02–2.92) than in non-smokers (*N*=177 cases; per log-unit HR, 1.11; 95% CI, 0.79–1.56), and a higher risk of lung cancer was found in non-smokers (*N*=222 cases; per log-unit HR, 1.57; 95% CI, 1.19–2.06) than in smokers (*N*=334 cases; per log-unit HR, 1.13; 95% CI, 0.91–1.39). However, no significant effect modification by BMI and smoking status was found. Stratification by GGT levels (⩽30 and >30 U l^−1^) revealed differential relationships between STG and overall cancer risk and lung cancer (data not shown).

## Discussion

In this large-scale cohort study, high STG concentrations were associated with higher overall cancer risk in women, but not in men. In men and women combined, STG concentrations were related to high risk of lung, thyroid, and rectal cancer. In men, STG concentrations were associated inversely with prostate cancer, and in women they were associated positively with gynaecological cancers. Our findings regarding lung, rectal, and gynaecological cancers are consistent with data using dietary fat intake levels as exposure variable (Kushi and Giovannucci, 2002; Genkinger *et al*, 2006).

Our observation of a positive association between STG levels and rectal cancer is in line with previous findings in a cohort study among Icelanders (Tulinius *et al*, 1997). Further evidence for a relationship with STG comes from case–control studies on colorectal adenoma (Kono *et al*, 1990; Bird *et al*, 1996; Otani *et al*, 2006; Tabuchi *et al*, 2006), carcinoma *in situ* (Yamada *et al*, 1998), and from an *in-vitro* study (Tabuchi *et al*, 2008). It has been suggested that total cholesterol, STG, and plasma glucose are positively associated with colorectal cancer risk (Yamada *et al*, 1998). In our study, the association occurred adjusted for plasma glucose and total cholesterol concentrations. In men and women combined, we observed an association between STG concentration and rectal cancer risk, whereas no association was found for colon cancer, neither was any association found in sex-stratified analyses. Consistent with a study among Japanese-American men (Tsushima *et al*, 2005) and US prospective studies (Saydah *et al*, 2003; Ahmed *et al*, 2006), we did not find a relationship with colorectal cancer.

Our findings of high lung cancer risk among subjects with high STG concentrations are unique. In one study, an association between total cholesterol and lung cancer risk has been observed, suggesting a relationship between lipid metabolism and lung cancer risk (Hinds *et al*, 1983). In the Carotene and Retinol Efficacy Trial (CARET), among the participants receiving *β*-carotene and retinol, higher serum triglyceride concentrations were observed (Cartmel *et al*, 2005), suggesting a relationship between STG and lung cancer risk. As smoking is associated with higher STG concentrations (Brunzell, 2007), residual confounding due to smoking may contribute to the association between STG concentrations and lung cancer risk. In our study, however, the association persisted when the data set was limited to non-smokers, suggesting that factors other than smoking status may contribute to the observed association. The limited differentiation between missing smoking data and non-smoking status may have resulted in misclassification of smoking status. However, smoking information from follow-up visits for most of the participants was used to complement the baseline smoking status.

The positive association with thyroid cancer risk is in line with findings in a cohort study (Tulinius *et al*, 1997). It may be relevant that BMI was positively associated with thyroid cancer (Renehan *et al*, 2008).

In our study, STG concentrations were inversely associated with prostate cancer risk, in contrast to the reverse findings in a case–control study (Wuermli *et al*, 2005). However, in this clinical-based study, prostate cancers were compared with benign prostate hyperplasia, in which STG levels were lower than those in cancer cases. In large cohort studies in Norway and the United States, no association between STG and prostate cancer risk was found (Lund *et al*, 2006; Tande *et al*, 2006). The application of prostate-specific antigen contributes to heterogeneity of phenotype (Etzioni *et al*, 2002), which may have distorted the relationship with STG. In addition, an inverse relationship for NHL was observed in our study. Previous reports on cholesterol indicted that reverse causation may substantially contribute to the risk-lowering effect of high blood lipids (Rose and Shipley, 1980; Lim *et al*, 2007; Strasak *et al*, 2009).

Our observation that STG concentrations were positively associated with kidney cancer incidence in men (data not shown) contrasts with a recently published meta-analysis on BMI and renal cancer risk (Renehan *et al*, 2008). However, in our study, after adjusting for diastolic blood pressure, an established risk factor for renal cancer (per log-unit HR, 1.26; 95% CI, 0.95–1.68), the association was no longer statistically significant. For men and women combined, we found no statistically significant association between STG and kidney cancer risk.

Our observation of a positive association of STG concentrations with risk of gynaecological cancers (cervix, ovary, endometrial) is consistent with other studies (Tulinius *et al*, 1997; Cust *et al*, 2007). In one study, increasing triglyceride and glucose concentrations were associated with increased endometrial cancer risk (Cust *et al*, 2007). Our findings on cervical cancer are in line with those in a cohort study (Tulinius *et al*, 1997). For breast cancer, inconsistent results have been reported from a nested case–control study (Agnoli *et al*, 2009) and from cohort studies (Vatten and Foss, 1990; Furberg *et al*, 2004).

These associations with gynaecological cancer raise a question with regard to the involvement of oestrogens, which are considered to stimulate hepatic triglyceride secretion (Sattler *et al*, 2005), as confirmed by studies on hormone replacement therapy (Rossouw *et al*, 2008; Sowers *et al*, 2008).

High STG concentrations may reflect other metabolic aspects that are procarcinogenic (McKeown-Eyssen, 1994). Associations between STG and plasma glucose levels are well established and hyperglycaemia is a risk factor for several cancers (Ashley and Kannel, 1974). In our study, however, we used fasting STG levels and adjusted for plasma glucose levels to control for confounding by glucose levels. Inflammation is another potential mechanism by which hypertriglyceridemia is associated with cancer risk (Esteve *et al*, 2005; Kundu and Surh, 2008). STG concentrations may be linked to colorectal cancer risk by bile acid excretion, circulation hormones, or energy supply to neoplastic cells (McKeown-Eyssen, 1994).

A limitation of our study is the lack of information on such potential risk factors as alcohol consumption and physical activity. However, the results of our multivariate models adjusted for GGT concentrations may be considered as a proxy variable for alcohol intake (Whitehead *et al*, 1978). In addition, no information on medication history (for example, on lipid-lowering drugs or hormones) was available, which may have affected the associations observed. Among women, the effect of STG may be overestimated because of residual confounding by exogenous hormones, whereas for lipid-lowering medications, the opposite could be relevant.

Undocumented measurement variation in STG concentrations during the study period may also have affected our results, but we assume that these were minor, as we used fasting STG levels. The strengths of our study are large sample size, prospective design, length of follow-up, and standardised examinations by trained physicians. It is relevant that the study population is relatively young and healthy.

Overall, STG concentrations were positively associated with the risk of lung, thyroid, and rectal cancers, but inversely with NHL risk. Prostate cancer risk was inversely associated with STG concentrations, whereas positive associations were found with renal cancer among men and with gynaecological cancers among women. Our results suggest that STG concentrations are involved in the pathogenesis of several cancer sites.

## Figures and Tables

**Table 1:** Characteristics of the study population (*N*=156 153) by quartile of serum triglyceride (STG) concentration

	**All**	**1st quartile**	**2nd quartile**	**3rd quartile**	**4th quartile**
Triglycerides (mg 100 ml^−1^), median (Q1, Q3)	104.0 (74.0, 152.0)	61.0 (52.0, 68.0)	88.0 (81.0, 96.0)	124.0 (114.0, 137.0)	207.0 (175.0, 269.0)
Total cholesterol (mg 100 ml^−1^), mean (s.d.)	213.7 (45.8)	188.8 (36.3)	206.1 (39.2)	220.0 (41.7)	240.9 (48.5)
Glucose (mg 100 ml^−1^), mean (s.d.)	87.2 (22.6)	83.7 (16.3)	85.0 (20.0)	87.2 (21.0)	93.0 (31.2)
Gamma-glutamyltransferase (GGT) (U l^−1^), median (Q1, Q3)	12.0 (8.0, 18.0)	9.0 (7.0, 13.0)	11.0 (8.0, 15.0)	12.0 (9.0, 19.0)	18.0 (12.0, 29.0)
Age (years), mean (s.d.)	41.8 (15.1)	37.2 (13.0)	40.8 (15.1)	43.4 (15.9)	50.0 (14.9)
BMI (kg m^−2^), mean (s.d.)	24.7 (4.2)	22.9 (3.4)	24.1 (3.9)	25.2 (4.3)	30.0 (4.3)
Current smokers (%)	25.2	20.4	23.7	26.1	30.8
Occupational status: white collar (%)	54.3	58.3	56.1	53.3	49.3

**Table 2:** Correlation of serum triglyceride (STG) concentrations with other clinical measures in the study population

**Covariates**	**Correlation coefficients** a	***P*-value**
BMI (kg m^−2^)	0.30	<0.001
Total cholesterol (mg 100 ml^−1^)	0.41	<0.001
Glucose (mg 100 ml^−1^)	0.14	<0.001
Gamma-glutamyltransferase (GGT) (U l^−1^)	0.32	<0.001

a Age- and sex- adjusted partial correlation coefficients, STG, and GGT were log transformed.

**Table 3:** Hazard ratios (HR with 95% CI) and numbers of cases for cancers by site and STG quartiles^a^

**Cancer site of both sexes ICD-10 codes**	**Total no. of cases**	**1st quartile**	**2nd quartile**	**3rd quartile**	**4th quartile**	**Total per log-unit increase**	***P*-value for log unit increase**
Thyroid C73	(101)	(16) 1.00	(25) 1.55 (0.82–3.00)	(30) 1.84 (0.97–3.48)	(30) 1.96 (1.00–3.84)	1.16 (0.76–1.76)	0.492
Plasmacytoma C90	(73)	(7) 1.00	(19) 1.88 (0.74–4.78)	(16) 1.37 (0.52–3.59)	(31) 2.11 (0.82–5.43)	1.39 (0.84–2.31)	0.204
NHL C82–C85	(219)	(42) 1.00	(61) 0.99 (0.66–1.49)	(54) 0.71 (0.46–1.07)	(62) 0.68 (0.43–1.07)	0.71 (0.52–0.97)	0.033
Stomach C16	(315)	(41)	(80) 1.32 (0.90–1.95)	(83) 1.22 (0.83–1.82)	(111) 1.45 (0.97–2.17)	1.17 (0.92–1.49)	0.208
Pancreatic C25	(162)	(24) 1.00	(35) 0.99 (0.58–1.69)	(39) 0.86 (0.50–1.47)	(64) 1.19 (0.70–2.05)	1.25 (0.90–1.75)	0.188
Colon C18	(600)	(81) 1.00	(122) 0.93 (0.70–1.23)	(175) 1.05 (0.80–1.39)	(222) 1.08 (0.81–1.43)	1.06 (0.88–1.26)	0.547
Rectal C19/20	(273)	(32) 1.00	(69) 1.47 (0.95–2.26)	(69) 1.25 (0.81–1.94	(103) 1.56 (1.00–2.44)	1.20 (0.92–1.55)	0.184
Bladder C67	(158)	(24) 1.00	(36) 1.50 (0.48–4.71)	(59) 0.73 (0.22–2.46)	(39) 1.42 (0.45–4.43)	1.03 (0.98–1.09)	0.218
Kidney C64	(216)	(35) 1.00	(40) 0.81 90.51–1.280	(51) 0.88 (0.57–1.38)	(90) 1.27 (0.81–1.97)	1.27 (0.95–1.69)	0.105
Lung C34	(650)	(86) 1.00	(128) (1.12 0.85–1.48)	(179) (1.43 1.09–1.87)	(257) (1.94 1.47–2.54)	1.50 (1.28–1.75)	<0.0001
							
*Men*							
Prostate C61	(1484)	(304) 1.00	(397) 0.94 (0.80–1.09)	(470) 0.87 (0.75–1.02)	(353) 0.67 (0.56–0.80)	0.80 (0.72–0.90)	<0.001
							
*Women*							
Breast C50 ⩽50 years	(510)	(127) 1.00	(157) 1.25 (0.98–1.59)	(136) 1.23 (0.95–1.59)	(90) 0.95 (0.70–1.28)	0.92 (0.74–1.15)	0.455
Breast >50 years	(694)	(75) 1.00	(135) 0.93 (0.70- 1.24)	200 1.00 (0.76–1.32)	284 1.05 (0.79–1.39)	1.09 (0.91–1.30)	0.352
Cervical C53	(70)	(12) 1.00	(17) 1.48 (0.69–3.19)	(18) 1.52 (0.70–3.34)	(23) 2.00 (0.89- 4.50)	1.74 (1.03–2.95	0.038
Endometrium C54	(236)	(22) 1.00	(50) 1.38 (0.83–2.30)	(54) 1.11 (0.66–1.86)	(110) 1.61 (0.97–2.67)	1.22 (0.90–1.65)	0.206
Ovarian C56	(123)	(16)	(29) 1.57 (0.81–3.04)	(39) 1.75 (0.91–3.37)	(39) 1.43 (0.71–2.85)	1.13 (0.74–1.74)	0.576
Gynaecological (C53, 54, 56)	(429)	(50) 1.00	(96) 1.45 (1.01- 2.07)	(111) 1.35 (0.94–1.93)	(172) 1.62 (1.13–2.33)	1.26 (1.01–1.58)	0.042

a Adjusted for BMI (kg m^−2^, continuous), GGT (continuous), serum glucose (continuous), total cholesterol concentration (continuous), smoking status, and occupational status.

## References

[bib1] Agnoli C, Berrino F, Abagnato CA, Muti P, Panico S, Crosignani P, Krogh V (2009) Metabolic syndrome and postmenopausal breast cancer in the ORDET cohort: a nested case-control study. Nutr Metab Cardiovasc Dis (E-pub ahead of print 8 April 2009) 10.1016/j.numecd.2009.02.006 PMC2819536 19361966

[bib2] Ahmed RL, Schmitz KH, Anderson KE, Rosamond WD, Folsom AR (2006) The metabolic syndrome and risk of incident colorectal cancer. Cancer 107: 28–36 16721800 10.1002/cncr.21950

[bib3] Ashley Jr FW, Kannel WB (1974) Relation of weight change to changes in atherogenic traits: the Framingham Study. J Chronic Dis 27: 103–114 4830839 10.1016/0021-9681(74)90079-4

[bib4] Bianchini F, Kaaks R, Vainio H (2002) Overweight, obesity, and cancer risk. Lancet Oncol 3: 565–574 12217794 10.1016/s1470-2045(02)00849-5

[bib5] Bird CL, Ingles SA, Frankl HD, Lee ER, Longnecker MP, Haile RW (1996) Serum lipids and adenomas of the left colon and rectum. Cancer Epidemiol Biomarkers Prev 5: 607–612 8824362

[bib6] Brunzell JD (2007) Clinical practice. Hypertriglyceridemia. N Engl J Med 357: 1009–1017 17804845 10.1056/NEJMcp070061

[bib7] Calle EE, Kaaks R (2004) Overweight, obesity and cancer: epidemiological evidence and proposed mechanisms. Nat Rev Cancer 4: 579–591 15286738 10.1038/nrc1408

[bib8] Cartmel B, Dziura J, Cullen MR, Vegso S, Omenn GS, Goodman GE, Redlich CA (2005) Changes in cholesterol and triglyceride concentrations in the Vanguard population of the Carotene and Retinol Efficacy Trial (CARET). Eur J Clin Nutr 59: 1173–1180 16015255 10.1038/sj.ejcn.1602229

[bib9] Cust AE, Kaaks R, Friedenreich C, Bonnet F, Laville M, Tjonneland A, Olsen A, Overvad K, Jakobsen MU, Chajes V, Clavel-Chapelon F, Boutron-Ruault MC, Linseisen J, Lukanova A, Boeing H, Pischon T, Trichopoulou A, Christina B, Trichopoulos D, Palli D, Berrino F, Panico S, Tumino R, Sacerdote C, Gram IT, Lund E, Quiros JR, Travier N, Martinez-Garcia C, Larranaga N, Chirlaque MD, Ardanaz E, Berglund G, Lundin E, Bueno-de-Mesquita HB, van Duijnhoven FJ, Peeters PH, Bingham S, Khaw KT, Allen N, Key T, Ferrari P, Rinaldi S, Slimani N, Riboli E (2007) Metabolic syndrome, plasma lipid, lipoprotein and glucose levels, and endometrial cancer risk in the European Prospective Investigation into Cancer and Nutrition (EPIC). Endocr Relat Cancer 14: 755–767 17914105 10.1677/ERC-07-0132

[bib10] Despres JP, Lemieux I (2006) Abdominal obesity and metabolic syndrome. Nature 444: 881–887 17167477 10.1038/nature05488

[bib11] Esteve E, Ricart W, Fernandez-Real JM (2005) Dyslipidemia and inflammation: an evolutionary conserved mechanism. Clin Nutr 24: 16–31 15681098 10.1016/j.clnu.2004.08.004

[bib12] Etzioni R, Penson DF, Legler JM, di Tommaso D, Boer R, Gann PH, Feuer EJ (2002) Overdiagnosis due to prostate-specific antigen screening: lessons from US prostate cancer incidence trends. J Natl Cancer Inst 94: 981–990 12096083 10.1093/jnci/94.13.981

[bib13] Furberg AS, Veierod MB, Wilsgaard T, Bernstein L, Thune I (2004) Serum high-density lipoprotein cholesterol, metabolic profile, and breast cancer risk. J Natl Cancer Inst 96: 1152–1160 15292387 10.1093/jnci/djh216

[bib14] Genkinger JM, Hunter DJ, Spiegelman D, Anderson KE, Beeson WL, Buring JE, Colditz GA, Fraser GE, Freudenheim JL, Goldbohm RA, Hankinson SE, Koenig KL, Larsson SC, Leitzmann M, McCullough ML, Miller AB, Rodriguez C, Rohan TE, Ross JA, Schatzkin A, Schouten LJ, Smit E, Willett WC, Wolk A, Zeleniuch-Jacquotte A, Zhang SM, Smith-Warner SA (2006) A pooled analysis of 12 cohort studies of dietary fat, cholesterol and egg intake and ovarian cancer. Cancer Causes Control 17: 273–285 16489535 10.1007/s10552-005-0455-7

[bib15] Gotto Jr AM (1998) Triglyceride: the forgotten risk factor. Circulation 97: 1027–1028 9531247 10.1161/01.cir.97.11.1027

[bib16] Hinds MW, Kolonel LN, Lee J, Hankin JH (1983) Dietary cholesterol and lung cancer risk among men in Hawaii. Am J Clin Nutr 37: 192–193 6823881 10.1093/ajcn/37.2.192

[bib17] Kahn HS, Valdez R (2003) Metabolic risks identified by the combination of enlarged waist and elevated triacylglycerol concentration. Am J Clin Nutr 78: 928–934 14594778 10.1093/ajcn/78.5.928

[bib18] Khovidhunkit W, Kim MS, Memon RA, Shigenaga JK, Moser AH, Feingold KR, Grunfeld C (2004) Effects of infection and inflammation on lipid and lipoprotein metabolism: mechanisms and consequences to the host. J Lipid Res 45: 1169–1196 15102878 10.1194/jlr.R300019-JLR200

[bib19] Kono S, Ikeda N, Yanai F, Yamamoto M, Shigematsu T (1990) Serum lipids and colorectal adenoma among male self-defence officials in northern Kyushu, Japan. Int J Epidemiol 19: 274–278 2376436 10.1093/ije/19.2.274

[bib20] Kundu JK, Surh YJ (2008) Inflammation: gearing the journey to cancer. Mutat Res 659: 15–30 18485806 10.1016/j.mrrev.2008.03.002

[bib21] Kushi L, Giovannucci E (2002) Dietary fat and cancer. Am J Med 113(Suppl 9B): 63S–70S 12566141 10.1016/s0002-9343(01)00994-9

[bib22] Langsted A, Freiberg JJ, Nordestgaard BG (2008) Fasting and nonfasting lipid levels. Influence of normal food intake on lipids, lipoproteins, apolipoproteins, and cardiovascular risk prediction. Circulation 118(20): 2047–2056 18955664 10.1161/CIRCULATIONAHA.108.804146

[bib23] Lim U, Gayles T, Katki HA, Stolzenberg-Solomon R, Weinstein SJ, Pietinen P, Taylor PR, Virtamo J, Albanes D (2007) Serum high-density lipoprotein cholesterol and risk of non-Hodgkin's lymphoma. Cancer Res 67: 5569–5574 17522388 10.1158/0008-5472.CAN-07-0212

[bib24] Lund HL, Wisloff TF, Holme I, Nafstad P (2006) Metabolic syndrome predicts prostate cancer in a cohort of middle-aged Norwegian men followed for 27 years. Am J Epidemiol 164: 769–774 16952929 10.1093/aje/kwj284

[bib25] McKeown-Eyssen G (1994) Epidemiology of colorectal cancer revisited: are serum triglycerides and/or plasma glucose associated with risk? Cancer Epidemiol Biomarkers Prev 3: 687–695 7881343

[bib26] Oberaigner W, Vittadello F (2006) Cancer mapping in Alpine Regions 1996–2000. Pro literature Verlag: Mammendarf

[bib27] Otani T, Iwasaki M, Ikeda S, Kozu T, Saito H, Mutoh M, Wakabayashi K, Tsugane S (2006) Serum triglycerides and colorectal adenoma in a case-control study among cancer screening examinees (Japan). Cancer Causes Control 17: 1245–1252 17111255 10.1007/s10552-006-0065-z

[bib28] Parkin DM, Whelan SL, Ferlay J, Teppo L, Thomas DB, all at the International Agency for Research on Cancer (2003) Cancer Incidence in Five Continents. IARC: Lyon, France

[bib29] Rapp K, Schroeder J, Klenk J, Stoehr S, Ulmer H, Concin H, Diem G, Oberaigner W, Weiland SK (2005) Obesity and incidence of cancer: a large cohort study of over 145 000 adults in Austria. Br J Cancer 93: 1062–1067 16234822 10.1038/sj.bjc.6602819 PMC2361672

[bib30] Rapp K, Schroeder J, Klenk J, Ulmer H, Concin H, Diem G, Oberaigner W, Weiland SK (2006) Fasting blood glucose and cancer risk in a cohort of more than 140 000 adults in Austria. Diabetologia 49: 945–952 16557372 10.1007/s00125-006-0207-6

[bib31] Renehan AG, Tyson M, Egger M, Heller RF, Zwahlen M (2008) Body-mass index and incidence of cancer: a systematic review and meta-analysis of prospective observational studies. Lancet 371: 569–578 18280327 10.1016/S0140-6736(08)60269-X

[bib32] Rose G, Shipley MJ (1980) Plasma lipids and mortality: a source of error. Lancet 1: 523–526 6102243 10.1016/s0140-6736(80)92775-0

[bib33] Rossouw JE, Cushman M, Greenland P, Lloyd-Jones DM, Bray P, Kooperberg C, Pettinger M, Robinson J, Hendrix S, Hsia J (2008) Inflammatory, lipid, thrombotic, and genetic markers of coronary heart disease risk in the women's health initiative trials of hormone therapy. Arch Intern Med 168: 2245–2253 19001202 10.1001/archinte.168.20.2245 PMC2726792

[bib34] Sarwar N, Danesh J, Eiriksdottir G, Sigurdsson G, Wareham N, Bingham S, Boekholdt SM, Khaw KT, Gudnason V (2007) Triglycerides and the risk of coronary heart disease: 10 158 incident cases among 262 525 participants in 29 Western prospective studies. Circulation 115: 450–458 17190864 10.1161/CIRCULATIONAHA.106.637793

[bib35] Sattler AM, Soufi M, Maisch B, Schaefer JR (2005) Lipids and lipoproteins in women. Herz 30: 368–374 16132239 10.1007/s00059-005-2708-3

[bib36] Saydah SH, Platz EA, Rifai N, Pollak MN, Brancati FL, Helzlsouer KJ (2003) Association of markers of insulin and glucose control with subsequent colorectal cancer risk. Cancer Epidemiol Biomarkers Prev 12: 412–418 12750235

[bib37] Sowers MR, Randolph Jr J, Jannausch M, Lasley B, Jackson E, McConnell D (2008) Levels of sex steroid and cardiovascular disease measures in premenopausal and hormone-treated women at midlife: implications for the ‘timing hypothesis’. Arch Intern Med 168: 2146–2153 18955645 10.1001/archinte.168.19.2146 PMC2727614

[bib38] Strasak AM, Pfeiffer RM, Brant LJ, Rapp K, Hilbe W, Oberaigner W, Lang S, Borena W, Concin H, Diem G, Ruttmann E, Glodny B, Pfeiffer KP, Ulmer H (2009) Time-dependent association of total serum cholesterol and cancer incidence in a cohort of 172 210 men and women: a prospective 19-year follow-up study. Ann Oncol 20(6): 1113–1120 19164459 10.1093/annonc/mdn736 PMC2685450

[bib39] Strasak AM, Pfeiffer RM, Klenk J, Hilbe W, Oberaigner W, Gregory M, Concin H, Diem G, Pfeiffer KP, Ruttmann E, Ulmer H (2008a) Prospective study of the association of gamma-glutamyltransferase with cancer incidence in women. Int J Cancer 123: 1902–1906 18688855 10.1002/ijc.23714

[bib40] Strasak AM, Rapp K, Brant LJ, Hilbe W, Gregory M, Oberaigner W, Ruttmann E, Concin H, Diem G, Pfeiffer KP, Ulmer H (2008b) Association of gamma-glutamyltransferase and risk of cancer incidence in men: a prospective study. Cancer Res 68: 3970–3977 18483283 10.1158/0008-5472.CAN-07-6686 PMC5955388

[bib41] Tabuchi M, Kitayama J, Nagawa H (2006) Hypertriglyceridemia is positively correlated with the development of colorectal tubular adenoma in Japanese men. World J Gastroenterol 12: 1261–1264 16534881 10.3748/wjg.v12.i8.1261 PMC4124439

[bib42] Tabuchi M, Kitayama J, Nagawa H (2008) Hyperglycemia and hypertriglyceridemia may associate with the adenoma-carcinoma transition in colorectal epithelial cells. J Gastroenterol Hepatol 23: 985–987 17683487 10.1111/j.1440-1746.2007.05072.x

[bib43] Tande AJ, Platz EA, Folsom AR (2006) The metabolic syndrome is associated with reduced risk of prostate cancer. Am J Epidemiol 164: 1094–1102 16968859 10.1093/aje/kwj320

[bib44] Tsushima M, Nomura AM, Lee J, Stemmermann GN (2005) Prospective study of the association of serum triglyceride and glucose with colorectal cancer. Dig Dis Sci 50: 499–505 15810632 10.1007/s10620-005-2464-5

[bib45] Tulinius H, Sigfusson N, Sigvaldason H, Bjarnadottir K, Tryggvadottir L (1997) Risk factors for malignant diseases: a cohort study on a population of 22 946 Icelanders. Cancer Epidemiol Biomarkers Prev 6: 863–873 9367058

[bib46] Ulmer H, Kelleher C, Diem G, Concin H (2004) Why Eve is not Adam: prospective follow-up in 149 650 women and men of cholesterol and other risk factors related to cardiovascular and all-cause mortality. J Womens Health (Larchmt) 13: 41–53 15006277 10.1089/154099904322836447

[bib47] Vatten LJ, Foss OP (1990) Total serum cholesterol and triglycerides and risk of breast cancer: a prospective study of 24 329 Norwegian women. Cancer Res 50: 2341–2346 2317820

[bib48] Whitehead TP, Clarke CA, Whitfield AG (1978) Biochemical and haematological markers of alcohol intake. Lancet 1: 978–981 76902 10.1016/s0140-6736(78)90261-1

[bib49] World Health Organisation (1998) Obesity: Preventing and Managing the Global Epidemic. Report of a WHO Consultation on Obesity. WHO: Geneva 11234459

[bib50] World Health Organization (2008) International Classification of Diseases (ICD). WHO: Geneva. Available at http://www.who.int/classifications/icd/en

[bib51] Wuermli L, Joerger M, Henz S, Schmid HP, Riesen WF, Thomas G, Krek W, Cerny T, Gillessen S (2005) Hypertriglyceridemia as a possible risk factor for prostate cancer. Prostate Cancer Prostatic Dis 8: 316–320 16158078 10.1038/sj.pcan.4500834

[bib52] Yamada K, Araki S, Tamura M, Sakai I, Takahashi Y, Kashihara H, Kono S (1998) Relation of serum total cholesterol, serum triglycerides and fasting plasma glucose to colorectal carcinoma *in situ*. Int J Epidemiol 27: 794–798 9839735 10.1093/ije/27.5.794

